# Expression of vascular infarction-related molecules after anti-vascular endothelium growth factor treatment for diabetic macular edema

**DOI:** 10.1038/s41598-019-48869-9

**Published:** 2019-08-26

**Authors:** Masahiko Sugimoto, Yasuko Wakamatsu, Ryohei Miyata, Takayasu Nunome, Yumiho Tenma, Hisashi Matsubara, Mineo Kondo, Hideo Wada, Kaname Nakatani

**Affiliations:** 1 Department of Ophthalmology, Mie University Graduate School of Medicine, 2-174, Edobashi, Tsu, 514-8507 Japan; 2 Department of Molecular and Laboratory Medicine, Mie University Graduate School of Medicine, 2-174, Edobashi, Tsu, 514-8507 Japan

**Keywords:** Retinal diseases, Endocrinology

## Abstract

To determine whether an intravitreal injection of anti-vascular endothelial growth factor (anti-VEGF) in eyes with diabetic macular edema (DME) affects the vascular infarction-related molecules (VIRMs). Nineteen eyes with DME were treated with 0.5 mg of intravitreal ranibizumab (IVR), and 22 eyes with DME were treated with 2 mg of intravitreal aflibercept (IVA). Blood was collected before, 1 week and 1 month after the injections. Aqueous humor was collected before and 1 month after the injections. The concentration of the VIRMs (cardiac myoglobin, cardiac troponin, intercellular adhesion molecule, monocyte chemotactic protein-1, matrix metalloproteinase-8, placental growth factor [PlGF], tenascin-C, tissue inhibitor of metalloproteinase-1, thrombospondin-2, vascular cell adhesion molecule-1, and VEGF) were determined by the multiplex assay. After the single injection of both types of anti-VEGF agents, the concentration of aqueous VEGF decreased significantly (*P* < 0.01). The plasma VEGF was reduced significantly at 1 week after the IVA (93.7 ± 17.6 to 39.5 ± 11.6 pg/ml; *P* < 0.01) but no significant change was seen after IVR (120.2 ± 11.3 to 137.4 ± 17.7 pg/ml). No significant changes were detected for the other VIRMs in the plasma and aqueous. A single intravitreal injection of anti-VEGF for DME does not significantly affect the concentration of several VIRMs.

## Introduction

Diabetic retinopathy (DR) is a common vision-threatening complication of diabetes mellitus, and it is present in over one-half of patients with diabetes mellitus of over 20 years duration^1^. Diabetic macular edema (DME) is a severe complication of DR that mainly affects the central vision^2^. Molecular biological studies have shown that vascular endothelial growth factor (VEGF) is involved in the onset and progression of DME^3–5^, and clinical studies have shown that anti-VEGF therapy can resolve the DME. Currently, anti-VEGF therapy has become the first line treatment for DME because it improves the visual function, and the improvement is maintained for long periods^6,7^. Three anti-VEGF agents are available; bevacizumab (Avastin^R^, Roche, Basal, Schweiz), ranibizumab (Lucentis^R^, Genentech, South San Francisco, CA, USA), and aflibercept (Eyelea^R^, Regeneron, Tarrytown, NY)^8–10^.

Generally, VEGF plays an important role in physiological vascular angiogenesis and restoring homeostasis after ischemia-reperfusion conditions due to a cerebral infarction or a myocardial infarction. This mechanism is important for tissues by protecting them from ischemic cell death. Although anti-VEGF agents are widely used for cancer treatment, their use can also lead to vascular damage such as arterial thromboembolism and re-occlusion of new vessels. In fact, VEGF suppression after systemic administration of anti-VEGF agents during cancer treatments has been associated with severe complications such as cardiovascular or arterial thrombotic complications^11,12^.

Fung *et al*. conducted an internet survey of 70 ophthalmological centers that had performed 7113 intravitreal injections of bevacizumab in 5228 patients. They reported that intravitreal injections of bevacizumab caused systemic complications in about 1 in 3000 patients^13^. Another study reported on an association between anti-VEGF treatment with bevacizumab or ranibizumab and systemic complications such as mortality, myocardial infarction, bleeding, and strokes^14^. A recent large-scale examination of the records of 57 919 patients reported a high incidence of adverse systemic events after anti-VEGF treatment (beacizumab and ranibizumab) for age-related macular degeneration^15^. Similar findings have also been reported for anti-VEGF treatment for DME^16^. Although evidence exists for such systemic complications, there is still no *in situ* evidence on the pathogenesis of these adverse events. On the contrary, a meta-analysis of 21 randomized cohort studies^17^ and comparisons of randomized controlled trials (RCTs) reported that the frequency of these complications was not so high^18,19^. In addition, because VEGF is reported to also enhance the progression of atherosclerotic plaques^20^ or is related to the activation of platelets^21^, its blockage can result in a reduction of vascular complication. This point contradicts with the side effects of anti-VEGF therapy. These findings are confusing for physicians in their use of anti-VEGF agents.

To have systemic effects, the intravitreally injected anti-VEGF agent must pass from the vitreous into the systemic circulation at high enough concentrations to be effective^22^. We have reported that unilateral intravitreal ranibizumab (IVR) injections do not affect the ocular circulation of the fellow eyes as determined by laser speckle flowgraphy^23^. In addition, there are many reports about the concentration of VEGF after anti-VEGF therapy for age-related macular degeneration and state that the systemic VEGF concentration after treatment differs for the different anti-VEGF agents^24–26^. However, these observations were only concerned with the VEGF activity. It is not still clear whether fluctuations of the VEGF levels were enough to cause systemic vascular infarctions or have significant clinical effects.

Many molecules have been shown to be associated with a risk of vascular infarctions including myocardial infarctions^27^. Cardiac troponin, cardiac myoglobin, creatinine kinase, and lactate dehydrogenase are well known molecules associated with cardiac infarctions. In addition, transient ischemic attacks are precursors of cerebral infarctions, and ICAM-1, IL-6, and c-related protein are also reported to be related to their onset^28^. Interestingly, these inflammatory factors including, ICAM-1 and IL-6 that are associated with these systemic complications, are reported to be related to the severity of DME^29^.

We hypothesized that these vascular infarction-related molecules (VIRMs) at downstream and other pathways of VEGF can be affected by anti-VEGF therapy. Thus, the purpose of this study is to evaluate and compare the expression of the VIRMs after an IVR or an IVA injection.

## Results

### Demographics of patients

Forty-one treatment-naïve eyes were studied. There were 19 eyes of 19 patients (14 men and 5 women) treated with intravitreal ranibizumab (IVR group) and 22 eyes of 22 patients (16 men and 6 women) treated with intravitreal aflibercept (IVA group) (Table 1). There was no significant difference in the sex distribution in the two groups (Chi square test, *P* = 0.78).

**Table 1:** Demographics of Patients.

	Age(yrs)	HbA1c(%)	BUN(mg/dl)	eGFR(m${\boldsymbol{\ell }}$/min/1.73 m^2^)	sBP (mmHg)	dBP(mmHg)
Ranibuzumab	65.8 ± 12.8	7.0 ± 1.0	25.6 ± 15.1	56.4 ± 28.5	141.4 ± 13.5	76.9 ± 11.2
Aflibercept	67.0 ± 6.9	7.1 ± 1.2	18.9 ± 6.4	64.9 ± 24.6	142.1 ± 20.9	73.2 ± 12.4
*p*-value	0.53	0.84	0.15	0.26	0.75	0.49

Data are the means ± standard deviations. Un-paired t-test was used to determine the significance between the groups,

BUN: blood urea nitrogen.

GFR: glomerular filtration rate.

sBP: Systolic blood pressure.systolic?

dBP: Diastolic blood pressure.diastolic?

The demographics of the patients are shown in Table 1. There was no significant difference in the background data between the two groups. For the IVR group, 6 patients had moderate non-proliferative (PDR), 10 had severe non-PDR, and 3 had non-high risk PDR. For the IVA group, 9 patients had moderate non-PDR, 12 had severe non-PDR, and 1 had non-high risk PDR. There was no significant difference in the distribution between the two groups (*P* = 0.78, Chi square test). For the IVR group, 7 patients had received pan-retinal photocoagulation (PRP) and 12 had not received PRP. For the IVA group, 5 patients had received PRP and 17 had not received PRP (*P* = 0.32; Chi square test).

For the IVR group, 16 patients were treatment naïve and 3 patients had received other therapy for the DME but still met the inclusion criteria; the 3 patients had received a sub-Tenon injection of triamcinolone acetonide. For the IVA group, 17 patients were treatment naïve and 5 patients had received other therapy for the DME but still met the criteria; 4 of the patients received an injection of sub-Tenon’s triamcinolone acetonide and 1 patient had received an intravitreal bevacizumab injection. There was no significant difference between the two groups (*P* = 0.70; Chi square test).

### Level of expression of VEGF and PlGF in IVR and IVA groups

We compared the levels of expression of VEGF and PlGF during the treatment period. The VEGF levels in the aqueous was significantly decreased from 138.1 ± 25.8 pg/ml to 58.5 ± 42.7 pg/ml at 1 month in the IVR group (*P* < 0.05), and from 164.6 ± 28.3 pg/ml to 4.8 ± 0.9 pg/ml at 1 month in the IVA group (*P < *0.01) (Table 2) . The plasma level of VEGF was significantly decreased from 93.7 ± 17.6 pg/ml to 39.5 ± 11.6 pg/ml at 1 week (*P < *0.01) and to 64.3 ± 15.2 pg/ml at 1 month after the IVA injection *(P* < 0.05). However, no significant change was observed for the plasma level of VEGF in the IVR group; 120.2 ± 11.3 pg/ml before to 137.4 ± 17.7 pg/ml at 1 week, and 136.6 ± 13.0 pg/ml at 1 month after the IVR injection.

**Table 2:** Level of VEGF and PlGF in IVR group and IVA group

		Ranibuzumab			Aflibercept		
		*pre*	1*w*	1 *M*	*pre*	*1w*	*1 M*
VEGF (pg/ml)	AC	138.1 ± 25.8		58.5 ± 42.7*	164.6 ± 28.3		4.8 ± 0.9**
	Plasma	120.2 ± 11.3	137.4 ± 17.7	136.6 ± 13.0	93.7 ± 17.6	39.5 ± 11.6**	64.3 ± 15.2*
PlGF (pg/ml)	AC	5.8 ± 1.9		5.4 ± 0.9	5.1 ± 1.1		3.6 ± 1.0*
	Plasma	3.7 ± 0.5	3.4 ± 0.4	3.4 ± 0.5	4.0 ± 0.5	5.6 ± 0.5**	3.7 ± 0.4

Data are the means ± standard deviations. Non-repeated ANOVA was used to determine the significance of the correlation between the groups, *P < 0.05, **P < 0.01.

AC: anterior chamber.

VEGF: vascular endothelial growth factor.

PlGF: placental growth factor.

The PlGF level in the aqueous was 5.8 ± 1.9 pg/ml before, and it was 5.4 ± 0.9 at 1 month in the IVR group with no significance. However, the PlGF level was significantly decreased in the IVA group during treatment from 5.1 ± 1.1 pg/ml before to 3.6 ± 1.0 pg/ml at 1 month (*P* < 0.05). The plasma level of PlGF did not change significantly in the IVR group during treatment (3.7 ± 0.5 pg/ml before to 3.4 ± 0.4 pg/ml at 1 week, and 3.4 ± 0.5 pg/ml at 1 month; no significant). However, the PIGF level was significantly increased from 4.0 ± 0.5 pg/ml before to 5.6 ± 0.5 pg/ml at 1 week after the injection in the IVA group (*P* < 0.01). The level was not significant at one month after the treatment (3.7 ± 0.4 pg/ml at 1 month).

### All VIRMs levels in aqueous and plasma did not change significantly in IVR and IVA groups

The levels of the VIRMs in the aqueous and plasma did not change significantly after both IVR and IVA injections (Table 3).

**Table 3:** Levels of the VIRMs in IVR group and IVA group.

		Ranibuzumab			Aflibercept		
		*pre*	*1w*	*1M*	*pre*	*1w*	*1M*
cardiac toroponin (pg/ml)	AC	56.5 ± 11.9		66.1 ± 17.3	58.4 ± 14.3		60.7 ± 16.7
	Plasma	45.6 ± 14.5	53.1 ± 15.1	63.2 ± 19.7	38.9 ± 12.3	48.8 ± 14.0	52.2 ± 17.0
ICAM1 (pg/ml)	AC	2.7 ± 0.8		2.7 ± 1.0	3.7 ± 0.9		3.1 ± 0.7
	Plasma	517.7 ± 94.8	518.6 ± 95.9	538.9 ± 111.5	483.9 ± 60.6	451.7 ± 53.0	462.1 ± 55.3
IL-6 (pg/ml)	AC	8.1 ± 1.0		7.6 ± 1.2	10.2 ± 1.2		12.1 ± 2.3
	Plasma	7.2 ± 1.7	5.6 ± 1.2	3.4 ± 0.5	6.3 ± 1.8	5.6 ± 1.4	6.5 ± 1.7
MCP-1 (pg/ml)	AC	673.0 ± 60.1		688.9 ± 80.1	815.6 ± 61.8		894.0 ± 72.8
	Plasma	298.6 ± 17.2	308.8 ± 20.0	353.2 ± 31.5	354.0 ± 25.0	360.7 ± 34.6	360.8 ± 37.4
MMP-8	AC (pg/ml)	125.0 ± 20.8		95.8 ± 24.5	147.0 ± 31.1		146.1 ± 31.5
	Plasma(ng/ml)	3.5 ± 0.6	2.7 ± 0.3	3.4 ± 0.7	3.2 ± 0.5	3.0 ± 0.6	3.6 ± 1.0
TIMP-1	AC(ng/ml)	24.2 ± 3.0		22.0 ± 3.0	28.4 ± 2.8		33.2 ± 3.6
	Plasma(ng/ml)	102.0 ± 127.8	110.2 ± 13.2	93.1 ± 16.0	93.8 ± 10.1	90.2 ± 9.5	94.2 ± 10.7
VCAM-1	AC (pg/ml)	37.9 ± 6.4		34.4 ± 6.0	46.1 ± 6.0		47.6 ± 7.0
	Plasma(ng/ml)	1.2 ± 0.1	1.2 ± 0.1	1.2 ± 0.1	1.4 ± 0.1	1.3 ± 0.1	1.3 ± 1.0
cardiac myogrobin	AC (pg/ml)	385.4 ± 199.8		220.1 ± 67.3	349.0 ± 75.2		336.8 ± 64.6
	Plasma(ng/ml)	9.4 ± 1.2	9.5 ± 1.6	10.3 ± 1.4	9.1 ± 1.0	7.8 ± 9.9	8.4 ± 9.0
tenaacin-C	AC (pg/ml)	256.8 ± 41.3		241.6 ± 53.0	364.6 ± 59.9		366.5 ± 62.8
	Plasma(ng/ml)	13.7 ± 5.8	13.5 ± 0.6	13.1 ± 8.3	13.3 ± 4.5	12.9 ± 4.6	13.1 ± 5.0
TSP-2	AC (pg/ml)	564.3 ± 93.2		502.2 ± 111.6	696.4 ± 95.6		653.9 ± 99.8
	Plasma(ng/ml)	265.4 ± 30.2	263.0 ± 28.3	264.3 ± 30.4	269.7 ± 29.2	266.1 ± 26.6	278.6 ± 23.6

Data are the means ± standard deviations. Non-repeated ANOVA was used to determine the significance of the correlation between the groups. AC: anterior chamber.

## Discussion

The results showed that IVA or IVR injections did not cause a significant increase in the levels of the VIRMs though behaviors of VEGF and PlGF were similar to previous reports^24–26,30^. These findings support previous results from large clinical trials in DME patients that concluded that the frequency of these complications was not high^17–19^.

Diabetes is reported to be associated with increases in the risk of vascular infarctions such as coronary heart disease and ischemic stroke^31–33^. A more recent study reported that not only the status of diabetes, but both the severity of DR and its progression are determinants of the incidence of cardiovascular complications^34^. Thus, there are important relationships between diabetes and diabetic retinopathy (DR) and the risk of vascular infarctions.

Myocardial infarction is one of the severe complications of diabetes. The findings in 7604 patients with type 2 DM indicated that there was an increased risk of cardiovascular diseases that required medical attention^35^. This is especially important because some of them occur without the patient being aware of their occurrence. Clinically, asymptomatic vascular infarctions without any symptoms often become serious complications in diabetic patients. A higher incidence of painless transient myocardial infarction has been reported in diabetic patients^36^. Lacunar infarctions are well known as asymptomatic cerebral infarctions, and they have been described as small, deep cerebral infarcts resulting from occlusions of small penetrating cerebral arteries^37^. A review of 2859 autopsy reports disclosed that lacunar infarctions were present in 34% of the diabetic patients^38^. Headaches are typical signs of a stroke onset and are rare symptoms in lacunar infarction which is different from a major stroke^39^. Because diabetes is associated with such minor vascular infarctions without any symptoms, there is a possibility that these complications may be masked in DME patients including those patients who received anti-VEGF therapy. This needs to be considered when patients are treated with anti-VEGF agents.

The use of systemic bevacizumab to treat cancer patients is closely associated with an increased risk of developing venous or arterial thromboembolism^40,41^. Ziv-aflibercept (Zaltrap^R^, Regeneron, Tarrytown, NY) is similar to aflibercept and is used to treat colorectal cancer or DME as an off-label usage^42,43^. Though no systemic complications were reported with off-label use for DME, arterial thromboembolic events occur at a much higher rate in cancer patients treated with ziv-aflibercept than those patients not treated with ziv-aflibercept (see prescribing information of ziv-aflibercept, https://www.accessdata.fda.gov/drugsatfda_docs/label/2012/125418s000lbl.pdf). These findings warn clinicians about the possibility of complications caused by anti-VEGF therapy.

Generally, DME patients receive multiple anti-VEGF injections, and Avery *et al*. stated that caution is needed in treating DME patients especially those who require frequent anti-VEGF injections^16^. Even though our results indicated that intravitreal anti-VEGF therapy did not cause an elevation of the VIRMs, these are short-term results observed at 1 month. Because the half-life of aflibercept and its ability to decrease free VEGF, is prolonged with more intraocular injections^24^, there is a possibility that the effect of VEGF may differ between multiple injections and a single injection. But there is also controversy on whether free VEGF is detectable after multiple injections of IVR or IVA^44^. Thus, it is still not clear whether these changes can cause systemic complications. Our results do not necessarily mean that anti-VEGF agents are safe after multiple IVR or IVA. We need to be cautious in administering anti-VEGF agents for DME patients, and we also have to examine VIRMs after multiple injections to determine the answer to this question.

Analyzing the systemic measurements of PlGF after IVA, we found a significant systemic upregulation of PlGF though no significant changes were observed for IVR group. This is consistent with a previous report for patients with neovascular age-related macular degeneration who received IVA which reported significant systemic upregulation of PlGF as same throughout the 4-weeks^30^. And dual anti–VEGF-A/PlGF inhibition induced increased secretion of systemic PlGF in tumor-bearing and nontumor-bearing mice^45^. This could represent a host counter-regulatory response to antiangiogenic therapy with IVA. There exists contradiction with such behavior of PlGF after IVA and its mechanism is still under debate.

The coagulation-related molecules (CoRMs), including soluble fibrin (SF), fibrinogen degradation products (FDP), and D-dimer have been shown to be risk factors for vascular thromboembolism. The expressions of these molecules are elevated in deep vein thrombosis^46–48^. The results of earlier studies showed that the serum D-dimer level was increased after systemic injections of bevacizumab in patients at risk of thromboembolism although it did not change for patient receiving IVR^49^. We did not detect any significant changes of the CoRMs during both IVA and IVR groups (Supplementary Data). Because we used EDTA and not citrate, theophylline, adenosine, and dipyridamole (CTAD) as the anticoagulant for serum collection, it may have affected the results. Though CTAD is usually used to preserve platelets and prevent activation of coagulation^50^, there was a tendency for an increase of SF and FDP in the IVA group at 1-week post-injection although no significant change was observed in the IVR group. These findings imply that there exists a possibility of a progression of coagulation after anti-VEGF treatment for DME.

Many studies have reported that caution is needed in the use of IVR and IVA because of the differences in the activity of ranibizumab and aflibercept. The DRCR.net protocol-T compared the one-year efficacy of three anti-VEGF agents for DME and showed that aflibercept was more effective than the other agents especially on the initial decrease in vision^51^. The results from an animal experiment showed that aflibercept reduced the VEGF level for a longer period than ranibizumab^52^, and it was taken up by neuronal and RPE cells whereas ranibizumab permeated the retina through the intercellular spaces^53^. Finally, aflibercept has a different mode of action from the other two anti-VEGF agents by suppressing not only VEGF but also PlGF. On the other hand, aflibercept has neutralizing effects against another angiogenic factor, galectin-1, independently of VEGF-A^54^. Thus, there are differences between ranibizumab and aflibercept in their clinical effectiveness and molecular properties. Although we did not detect any differences between these two approved anti-VEGF agents, there is a possibility that aflibercept has other effects that accelerate vascular coagulation. There is a possibility that in case of patient with a poorly controlled or long-standing diabetes and previous history of ischemic attacks who have potential risk to cause systemic complication after anti-VEGF treatment, it would be more convenient to place agents with less systemic blockade of VEGF such as ranibizumab. However, due to our small sample size, we did not detect any significant differences between ranibizumab and aflibercept, and further investigations are needed.

There are some limitations in our study including the small sample size. Although there seems to be a significant increase in MCP-1 (Table 3) in the plasma after 1 month of ranibizumab treatment compared to the baseline, it was not significant. The small sample size may have affected these results. Second, the concentrations of the different molecules were not necessarily similar to that of previous studies. For example, the baseline VEGF values for the IVR group was 17.0 pg/ml (Avery *et al*.^24^, serum sample), 19.7 pg/ml (Wang *et al*.^25^, plasma sample), 27.5 pg/ml (Wang *et al*.^25^, serum sample), and 245.7 pg/ml (Yoshida *et al*.^26^, plasma sample), and 120.2 pg/ml in our results (plasma sample). One of the reasons for these differences was because our quantification was performed using Multiplex assay and the other studies used enzyme-linked immunosorbent assays (ELISA). In general, the cytokine expression patterns have been analyzed by ELISA but that requires significant sample volumes and is limited to single analyte. We used a multiplex assay, and the advantages of this assay to ELISA system are higher throughput, smaller sample volume, and lower cost. There are good correlations between these two assays obtained for many molecules but not for all molecules. This is probably due to the antibody pairs and sample diluent composition^55,56^. Thus, it is difficult to compare the concentrations of our molecules and that of previous studies. But because our multiplex results were obtained under the same condition, it is possible to compare concentrations during the treatment as long as necessary in our cohort. In addition, we should consider conducting cellular or animal models for further confirmations. Third, 7 of 19 patients in the IVR group and 5 of the 22 patients in the IVA group had received PRP. Based on previous literature, there is a possibility that previous PRP treatments can influence the VEGF and VIRMs concentrations^57,58^.

And finally, our results do not truly support the safety of the anti-VEGF therapy. Also, VIRMs and CoRMs may cause mild tissue damage, and they do not reflect the possibility of severe infarctions. There are many other factors associated with vascular infarctions other than the VIRMs and CoRMs we evaluated. We did not evaluate MMP-2, MMP-9, C-reactive protein, serum neurofilament light chain, protein C, and protein S^28,59,60^. It is very difficult to evaluate all of these molecules because they may predict damages more sensitively, we have to check their concentration. The problem with these molecules including molecules evaluated in our study is that they can only be elevated by severe organ damage but they cannot predict the onset. In order to conclude that these molecules most likely do not induce any type of vascular infarctions, we need additional time points and experiments.

In conclusion, the results show that a single intravitreal injection of these two antiangiogenic agents for DME do not significantly affect the levels of VIRMs and CoRMs. However, we still need to pay attention to the general conditions in diabetic patients because of subclinical vascular damage, and it is still important to assess much more sensitive molecules as biomarkers of potential infarctions and systemic complications.

## Methods

The protocol of this study was approved by the Institutional Review Board of Mie University Hospital (No. 2831, approved 26, Dec. 2014), and all patients signed a written informed consent form before the study began. This prospective study was registered at http://www.umin.ac.jp (No. UMIN000021642, approved 03/Mar/2016). All methods were performed in accordance with the relevant guidelines and regulations.

This was a prospective, interventional case series that determined the levels of VIRMs in the blood and aqueous before and after the injection of IVA or IVR in patients with DME. This study was performed on patients examined between March 2015 and March 2016.

### Inclusion and exclusion criteria

Because ranibizumab and aflibercept are approved in Japan and bevacizumab is not, we used these two approved anti-VEGF agents for the DME treatment. The inclusion criteria were; (1) patients ≥20-years with type I or type II diabetes, (2) eyes with DR and DME and the diagnosis of DME was made by the clinical and spectral domain optical coherence tomographic (SD-OCT) findings, (3) DME involving the fovea and defined as a central macular thickness (CMT) ≥300 µm measured as the mean retinal thickness in the central 1 mm diameter circle in the OCT images.

Hematological analyses were done before the treatment to measure the level of hemoglobin A1c (HbA1c, NGSP, normal value 4.9–6.0%), blood urea nitrogen (BUN, normal value 8–20 mg/dl), and estimated glomerular filtration rate (eGFR, normal value 60–120 ml/min/1.73 m^2^). These were the indices for blood sugar level and renal function control. The blood pressure was also measured before and after the treatment. The stage of DR and previous PRP before treatment were obtained from the medical charts.

The exclusion criteria were; prior ocular surgery, macular laser photocoagulation, and intravitreal or sub-tenon injections of any drugs including anti-VEGF agents within 2 months of the beginning of this study. In addition, eyes with ocular inflammation, drusen, severe proliferative diabetic retinopathy, retinal hemorrhage which involved the intra- or sub-foveal spaces, an epiretinal membrane, prior pars plana vitrectomy, glaucoma, and media opacities that significantly affected the best-corrected visual acuity (BCVA) were excluded. Patients with uncontrolled systemic medical conditions or any history of thromboembolic events or ischemic diseases including myocardial infarction and cerebral infarction were also excluded. Patients who were being treated with anticoagulants such as aspirin, with hyper-coagulability or hypo-coagulability diseases, and being treated with systemic anti-VEGF agents, such as bevacizumab, for cancer were also excluded.

### Intravitreal anti-VEGF injection

Each eye received a single intravitreal injection of either 0.5 mg of ranibizumab (IVR; Lucentis^R^) or 2 mg of aflibercept (IVA, Eylea^R^) under topical anesthesia. The intravitreal injection was made with a 30-gauge needle that was inserted 4 mm posterior to the corneal limbus under sterile conditions. All patients received topical levofloxacin hydrate (1.5% Cravit ophthalmic solution^R^) after the anti-VEGF injection.

### Sample collection

Venous blood samples, plasma and serum, were collected from the patients before the first injection, at 1 week, and 1 month after the injection. The serum samples were collected in tubes containing EDTA as an anticoagulant. The samples were centrifuged 2000 x g for 5 min, and the supernatants were removed and immediately frozen and stored at −80 °C until assayed. Vitreous fluid was not collected because it can cause complications such as retinal detachments, we collected anterior chamber fluid. About 50 µl of aqueous sample was collected before the first injection and 1 month after the 2nd injection. The samples were frozen immediately and stored at −80 °C until assayed.

### Measurements of concentrations of vascular infarctions molecules (VIRMs)

The plasma concentrations of different VIRMs, e,g., cardiac myoglobin, cardiac troponin, intercellular adhesion molecule-1 (ICAM-1), interleuikin-6 (IL-6), monocyte chemotactic protein-1 (MCP-1), matrix metalloproteinase-8 (MMP-8), placental growth factor (PlGF), tenascin-C, tissue inhibitor of metalloproteinase-1 (TIMP-1), thrombospondin-2, vascular cell adhesion molecule-1 (VCAM-1), and VEGF were determined using the Luminex Multiplex Assay kit (Luminex Corporation, Austin, TX). Multianalyte profiling was performed with the Luminex-100 system and the XY Platform. Calibration microspheres were used for classification, reporter readings, and sheath fluid, and they were also purchased from Luminex Corporation. The acquired fluorescence data were analyzed by the Master Plex ^TM^ QT software (Ver. 1.2, Mirai Bio, Inc. San Bruno, CA). All analyses were performed according to the manufacturer’s instructions.

### Statistical analyses

All values are presented as the means ± standard deviations. The significance of the differences of the values was determined by two-way non-repeated ANOVA followed by Bonferroni post-hoc tests for the comparison of the means. Unpaired *t* tests were used to determine the significance of differences between two groups. Chi square tests were used to check the variance of the groups. Statistical significance was set at *P* < 0.05.

## Supplementary information

Levels of all CoRMs did not change significantly in IVR and IVA groups
