# Supplementary material for: Expression of vascular infarction-related molecules after anti-vascular endothelium growth factor treatment for diabetic macular edema
Source: Sci Rep. 2019 Aug 26;9:12373. doi: 10.1038/s41598-019-48869-9 (PMC6710265; doi:10.1038/s41598-019-48869-9)
Supplement: Supplementary file 1 — Levels of all CoRMs did not change significantly in IVR and IVA groups [file 41598_2019_48869_MOESM1_ESM.docx]

**Expression of vascular infarction-related molecules after**

**anti-vascular endothelium growth factor**

**treatment for diabetic macular edema**

Masahiko Sugimoto^1,3^, Yasuko Wakamatsu^1^, Ryohei Miyata^1^, Takayasu Nunome^1^, Yumiho Tenma^1^, Hisashi Matsubara^1^, Mineo Kondo^1^, Hideo Wada^2^, Kaname Nakatani^2^

^1^Department of Ophthalmology, Mie University Graduate School of Medicine

^2^Department of Molecular and Laboratory Medicine, Mie University Graduate School of Medicine

^3^Corresponding Author: Masahiko Sugimoto, MD, PhD, Department of Ophthalmology, Mie University Graduate School of Medicine. 2-174, Edobashi, Tsu, 514-8507, Japan.

Phone: +81-59-231-5027, Fax: +81-59-231-3036,

E-mail: sugimochi@clin.medic.mie-u.ac.jp

**Supplemental Information**

We also hypothesized that coagulation-related molecules (CoRMs) can also be affected. The serum levels of CoRMs, soluble fibrin (SF), fibrinogen degradation products (FDP), and D-dimer were measured by the latex agglutination method using Nanopia SF reagents, Nanopia FDP, and Nanopia D-dimer (Sekisui Medical, Tokyo, Japan), respectively. The levels of the CoRMs in the aqueous samples could not be determined because of the low volume of the aqueous collected.

**Levels of all CoRMs did not change significantly in IVR and IVA groups (supplementary table).**

We compared the concentration of SF, FDP, and D-dimer, types of CoRMs, in the serum samples after the IVR and IVA injections. The results showed that the levels of these 3 molecules did not change significantly during the experimental period in both the IVR and IVA groups. A tendency of SF and FDP to increase was observed more in the IVA group than in the IVR group (SF levels were 0.2 ± 0.3 μg/ml before, 0.1 ± 0.2 μg/ml at 1 week, and 0.2 ± 0.5 μg/ml at 1 month after IVR; 0.2 ± 0.2 μg/ml before, 0.3 ± 0.3 μg/ml at 1 week, and 0.3 ± 0.4 μg/ml at 1 month after IVA).The FDP level was 6.2 ± 2.9 µg/ml before treatment, 5.0 ± 1.6 µg/ml at 1 week, and 6.5 ± 2.9 µg at 1 month after IVR; 5.3 ± 1.8 µg/ml before, 7.5 ± 6.7 μg/ml at 1 week, and 5.1 ± 3.4 µg/ml at 1 month after IVA . No significant changes were observed in the expression levels of these molecules in both the IVR and IVA groups during the observation period.

**Supplemental Table. Levels of the CoRMs in IVR group and IVA group**

**Ranibuzumab Aflibercept**

**(μg/ml)** ***pre 1w 1M pre 1w 1M***

**SF**  0.2±0.3 0.1±0.2 0.2±0.5 0.2±0.2 0.3±0.3 0.3±0.4

**FDP** 6.2±2.9 5.0±1.6 6.5±2.9 5.3±1.8 7.5±6.7 5.1±3.4

**D-dimer** 2.8±1.8 2.0±1.0 2.9±1.6 2.1±1.0 2.1±0.9 2.1±2.1

Data are the means ± standard deviations. Non-repeated ANOVA was used to determine the significance of the correlation between the groups.

SF: soluble fibrin.

FDP: fibrin and fibrinogen degradation products.
